# Type D personality in Russian patients with cardiovascular disease: validity of the Russian DS14 (DS14-RU)

**DOI:** 10.1186/s12872-019-1056-9

**Published:** 2019-04-02

**Authors:** Georgiy Pushkarev, Johan Denollet, Vadim Kuznetsov, Viola Spek, Elena Yaroslavskaya

**Affiliations:** 1 Laboratory of Instrumental Diagnostics, Tyumen Cardiology Research Center, Tomsk National Research Medical Center, Russian Academy of Science, 111, Melnikaite Str, 625026 Tyumen, Russia; 2 Tyumen Cardiology Research Center, Tomsk National Research Medical Center, Russian Academy of Science, Tomsk, Russia; 3 Department of Medical and Clinical Psychology, CoRPS-Center of Research on Psychology in Somatic Diseases, Tilburg University, Tilburg, the Netherlands

**Keywords:** Type D personality, Reliability and validity, Psychosocial factors, Coronary artery disease

## Abstract

**Background:**

Type D personality is associated with unfavorable outcomes in patients with cardiovascular diseases (CVD). However, there is no valid Type D Scale in Russian language. The purpose of the study was to examine the factor structure of a new Russian version of 14-item Type D Scale (DS14-RU), and to evaluate the reliability and construct validity of the DS14-RU in clinical research.

**Methods:**

The study included 929 participants, 496 (53.4%) of which had coronary artery disease, 195 (21.0%) congestive heart failure, 84 (9.0%) arterial hypertension and 154 (16.6%) were relatively healthy volunteers. The mean age was 57.5 years, 565 (60.8%) participants were males. The respondents filled out an extended Russian version of the Type D scale and new DS14-RU, as well as the Hospital Anxiety and Depression Scale, Multidimensional Scale of Perceived Social Support, Reeder Stress Inventory, and State-Trait Personality Inventory.

**Results:**

The new Russian version of DS14-RU was internally consistent with Cronbach’s α = .80 for both the negative affectivity and social inhibition subscales. The prevalence of Type D personality, as measured with the DS14-RU, was 21.4% among patients with CVD, and 20.0% among relatively healthy participants. The mean scores for anxiety, depression, psychosocial stress and anger were significantly higher in patients with Type D personality and they had significantly lower levels of social support and curiosity.

**Conclusions:**

The new DS14-RU is consistent with the original DS14 in terms of reliability, factor structure and construct validity. The DS14-RU can be used for the reliable assessment of Type D in Russian-speaking respondents.

## Background

Previous studies have shown that Type D personality is a predictor of unfavorable outcomes in patients with different cardiovascular diseases (CVD), including coronary artery disease (CAD), history of myocardial infarction, congestive heart failure, peripheral arterial disease, and in patients who had undergone percutaneous coronary intervention (PCI) and heart transplantation [1, 2]. Moreover, Type D personality in these patients and in patients after coronary artery bypass grafting and cardiac resynchronisation therapy was associated with significant decrease in quality of life [2, 3]. Other studies have shown a tendency to unhealthy life style, non-completion of cardiac rehabilitation programs and poor compliance to treatment in patients with Type D personality [4, 5]. However, there are also studies that found no association between Type D personality and all-cause mortality [6, 7].

Considering these facts, assessment of Type D personality seems appropriate to identify patients at high risk of cardiovascular complications. For this purpose, the standard 14-item Type D Scale (DS14) was developed in Belgium and the Netherlands [8], which has subsequently been validated in many different countries including, for example, Denmark [9], Greece [10], Iceland [11], Poland [12], Taiwan [13], Korea [14], China [15], France [16], Germany [17], Iran [18], and Israel [19]. A large international study of 6222 cardiac patients from 22 different countries (Europe, USA, Canada and Australia) further confirmed the cross-cultural validity of the DS14 [20]. However, research in Asian countries showed that it was extremely rare for respondents from these countries to endorse a positive response to item #3 “*I often talk to strangers*”, causing a decrease in internal consistency and inflated scores of the social inhibition component [13–15].

In a previous study, we faced the same problem and this apparently means that Russian people are not accustomed to talk with strangers [21]. In other studies, this issue was addressed by replacing the original item #3 of the DS14 with an alternative social inhibition item that is more consistent with the cultural context of that country [13–15]. Therefore, the aim of this study was to evaluate of a new Russian version of the DS14 (DS14-RU) that includes an alternative social inhibition item #3, and to examine the prevalence of Type D personality and its association with anxiety and depression symptoms in Russian cardiac patients.

## Methods

### Participants

A total of 929 participants were included in the study, among them 496 participants (53.4%) with CAD and who had undergone PCI, 195 participants (21.0%) with congestive heart failure, 84 participants (9.0%) with arterial hypertension, and 154 participants (16.6%) who were relatively healthy volunteers selected from post-graduate students of Tyumen State Medical University. A total of 565 participants (60.8%) were males, and 364 (39.2%) were females. The age of the patients ranged from 21 to 90 years, mean age was 57.5 ± 12.7 years. Signed informed consent was obtained from all participants before enrolment in the study. The study was performed according to the Declaration of Helsinki and was approved by the local Ethics Committee.

### Assessment of Type D personality

For Type D personality identification, the standard Russian version of the 14-item Type D Scale (DS14) was used [22]. The DS14 questionnaire comprises two subscales: negative affectivity (NA) and social inhibition (SI), containing seven questions each. To express agreement/disagreement with each item, a 5-point Likert scale from 0 (false) to 4 (true) was used. Hence, the total scores for NA and SI subscales ranges from 0 to 28. If the score was ≥10 points on both subscales, Type D personality was diagnosed [8]. Validation of the Russian version of the questionnaire was performed earlier in the group of patients with CAD [21]. Performed exploratory factor analysis demonstrated a two-factor structure of the questionnaire, explaining 45% of the total variance (29.1% – first factor and 15.9% – second factor). The Cronbach’s α, related to internal consistency, was higher for NA scale (.78) than for SI scale (.74). Importantly, the corrected item-total correlation for item #3 “*I often talk to strangers*” (.21) was significantly lower than for the other 6 social inhibition items, and deletion of this item improved the internal consistency (Cronbach’s α) of the social inhibition subscale [21].

A previous study of the second author (JD) showed that the items “*I don’t like to have a lot of people around me*” and “*When I meet a lot of people, I get nervous*” were also good markers of social inhibition in Belgian respondents [23]. Therefore, these two items were included in an extended Russian version of the Type D scale (DS-Ex) that comprised 9 items of social inhibition, in addition to the regular 7 negative affectivity items. These two additional items, which reflect the ‘withdrawal’ facet of social inhibition [23], were considered as a candidate new item to replace the original item #3 of the DS14 in the Russian translation. Based on their psychometrical properties, we wanted to select the best alternative item to comprise a final 7-item SI subscale for the new Russian version of the DS14 (DS14-RU).

### Assessment of social support, anxiety, depression, stress and anger

To examine the construct validity of the DS14-RU, participants filled out questionnaires to assess their level of social support, anxiety, depression, psychosocial stress, anger, and curiosity.

To determine the amount of social support, the Multidimensional Scale of Perceived Social Support (MSPSS) was used. The Russian version of the MSPSS was validated and has a high internal consistency with Cronbach’s α between .90 and .91 [24]. To evaluate anxiety and depression levels, the Hospital Anxiety and Depression Scale (HADS), comprised of two equal subscales, was used. The HADS has been validated in many countries, and is considered a reliable questionnaire, with the Cronbach’s α ranging between .67 and .93 for both scales [25].

Psychosocial stress was measured by the Reeder Stress Inventory (RSI). Analysis of psychometric factors of the test revealed a one-factor structure with a high internal consistency (Cronbach’s α varied from .78 to .80) [26]. The State-Trait Personality Inventory (STPI) was used to measure dispositional/trait anger, anxiety, curiosity, and depression. The Russian version of the STPI has a high level of reliability, the Cronbach’s α ranged from .86 to .92 [27].

### Statistical analyses

To assess the internal consistency, Cronbach’s α, corrected item-total correlations (CITC) and mean inter-item correlations (MIIC) were calculated. A Cronbach’s α > .70, CITC at least .40 and MIIC in the range of .15 to .50 confirm appropriate consistency of the test [28, 29]. The temporal stability of the DS14-RU subscales was examined with Pearson’s correlation. For this purpose, 76 participants completed the DS14-RU once more within 3–4 months after the first examination.

To evaluate invariance of the scale and model-data fit, we randomly divided the participants into two approximate halves and conducted exploratory factor analysis (EFA) and confirmatory factor analysis (CFA) on each half, respectively. Testing the feasibility of using EFA was performed based on the Kaiser-Meyer-Olkin measure of sampling adequacy. The Bartlett’s sphericity test was used to test the null hypothesis of absence of correlation between variables in the general totality. EFA was performed using the principal component analysis with varimax rotation. The number of principal components was determined by means of minimum average partial criterion [30].

CFA was performed and the following values were calculated: the comparative fit index (CFI), the Tucker-Lewis index (TLI), the incremental fit index (IFI), the root mean-square error of approximation (RMSEA), the ratio of chi-squared test χ^2^ to the degree of freedom (χ^2^/*df*). The model was considered as consistent with the experimental data at the values of CFI, TLI and IFI ≥ 0.90, RMSEA less than 0.08 and χ^2^/*df* less than 5 [31, 32].

The construct validity of the DS14-RU and the MSPSS, HADS, RSI, STPI was examined using Pearson’s correlations.

The categorical variables were compared by means of the chi-square test (χ^2^), the continuous variable with normal distribution - by the Student’s t-test for independent samples. In case of non-normal variables, the Mann–Whitney nonparametric test was used.

The statistical analysis of the data was performed using IBM SPSS Version 21 and IBM SPSS AMOS Version 21.

## Results

The participant’s characteristics are presented in Table 1. Young women predominate in the group of relatively healthy volunteers, and older men predominate in the group of patients with CVD. Almost one third of patients in the CVD group had a higher education. The majority of participants were married. The prevalence of Type D personality, as measured with the DS14-RU was 21.4% among the patients with CVD, and 20% among the relatively healthy participants. In general, Type D personality was identified in 21.3% of the respondents.

**Table 1:** Demographic and clinical characteristics of participants

	Relatively healthy volunteers (*n* = 154)	Patients with CVD (*n* = 775)	p
Age, years	38.9 ± 11.7	60.7 ± 9.8	< 0.001
Male, %	14.8	70.2	< 0.001
Higher education, %	100.0	30.6	< 0.001
Married, %	68.2	75.7	0.03
Type D personality (DS14-RU)	20.0	21.4	NS
CAD, %	–	64.0	
CHF, %	–	25.2	
Hypertension, %	–	10.8	

*CVD* cardiovascular diseases, *DS14-RU* new Russian version of 14-item Type D Scale, *CAD*: coronary artery disease, *CHF* congestive heart failure, *NS* not significant

The internal consistency analysis for the different variants of the DS is presented in the Table 2. According to the data of this table, the CITC value for item #3 of the original DS14 scale was .21, which is less than the acceptable level of .40. Therefore, removal of this item (“*I often talk to strangers*”) from the original version of the DS14 led to an increase of Cronbach’s α from .74 to .77, and, consequently, to an increase of internal consistency of the test. Meanwhile, the CITC value of item #3 was .36 in the 9-item social inhibition measure of the DS-Ex, which was also below the threshold value of .40. The item “*I don’t like to have a lot of people around me*” of the DS-Ex also failed to meet the internal consistency criteria (CITC value .34). However, the DS-Ex item “*When I meet a lot of people, I get nervous*” had a high CITC value (.56) and removal of this item led to a decrease in Cronbach’s α from .79 to .76 in the DS-Ex.

**Table 2:** Reliability of the DS14 in Russian respondents: DS14, DS-Ex and DS14-RU

Item Content		Corrected Item-Total Correlations			Cronbach’s α in the case of item removal		
		DS14	DS-Ex	DS14-RU	DS14	DS-Ex	DS14-RU
DS14 Negative affectivity items					α = .78	α = .79	α = .80
2	I often make a fuss about unimportant things	.48	.47	.51	.77	.77	.79
4	I often feel unhappy	.51	.57	.60	.76	.75	.77
5	I am often irritated	.58	.46	.59	.74	.77	.77
7	I take a gloomy view of things	.48	.57	.52	.76	.76	.78
9	I am often in a bad mood	.52	.53	.58	.75	.76	.77
12	I often find myself worrying about something	.49	.48	.41	.76	.77	.80
13	I am often down in the dumps	.53	.62	.62	.75	.74	.76
DS14 Social inhibition items					α = .74	α = .79	α = .80
1	I make contact easily when I meet people	.50	.56	.45	.71	.77	.79
6	I often feel inhibited in social interactions	.41	.50	.58	.72	α = .77	.77
8	I find it hard to start a conversation	.55	.63	.60	.69	.75	.76
10	I am a closed kind of person	.57	.50	.57	.68	.77	.77
11	I would rather keep other people at a distance	.48	.50	.46	.71	.77	.79
14	When socializing, I don’t find the right things to talk about	.54	.47	.62	.69	.77	.76
*3*	*I often talk to strangers* ^a^	*.21*	*.36*	*–*	*.77*	*.79*	*–*
Extended Scale social inhibition items							
*15*	*I don’t like to have a lot of people around me* ^a^	*–*	*.34*	*–*	*–*	*0.79*	*–*
16	When I meet a lot of people, I get nervous^b^	–	.56	.48	–	0.76	.79

*DS14* standard 14-item Type D Scale, *DS-Ex* extended Russian version of the Type D scale, *DS14-RU* new Russian version of 14-item Type D Scale

^a^Social inhibition items that were not included in the final version of the Russian DS14

^b^New social inhibition item included in the Russian DS14 to replace the original item #3

Therefore, in the final Russian version of the DS14, the original social inhibition item #3 was replaced by the alternative item “*When I meet a lot of people, I get nervous*”, leading to an increase of Cronbach’s α from .74 to .80, and, consequently, to an increase of internal consistency of the 7-item social inhibition subscale of the DS14-RU. All 7 items of the NA subscale had high values of the CITC, both in the translation of the original DS14 and in the DS-Ex, confirming appropriate internal consistency of the original NA subscale, and indicating that any replacement of NA items was not required in the Russian version of the DS14. These data were confirmed by the DS14-RU analysis; the CITC value for all NA subscale questions was more than .40. Eventually, the Cronbach’s α for NA subscale of the DS14-RU was .80, which was similar to the SI subscale. The MIIC value for the NA subscale was .39, and for the SI subscale .37, confirming appropriate internal consistency of the resulting scale.

The DS14-RU had good test-retest reliability, within an average of 3.6 months the test-retest Pearson’s correlation coefficient for the NA subscale was .70 and for the SI subscale .71.

Values of the Kaiser-Meyer-Olkin test for sampling adequacy (0.87) and the Bartlett’s sphericity test (χ^2^(91) = 2283, *p* < 0.001) show that correlation matrix for the DS14-RU can be used for further factor analysis. The results of the factor analysis are presented in Table 3. The minimum average partial criterion was used for selection of the two principal components (factors) with the eigenvalues greater than 1, which could be designated as NA and SI. The 2-factor model explains 48.3% of the total variance (34.9% of the first factor and 13.4% of the second factor). Table 3 shows that factor loading and communality (h^2^) for the questions in the two-factor solution ranged from .54 to .74 and from .36 to .61, respectively. Therefore, the two-factor solution is consistent with the theoretical conceptions of the structure.

**Table 3:** Final version of the Russian DS14 (DS14-RU): principal components matrix

Items		Factor 1	Factor 2	Communality (h^2^)
Negative affectivity				
2	I often make a fuss about unimportant things	.71	−.08	.51
4	I often feel unhappy	.70	.20	.53
5	I am often irritated	.69	.06	.47
7	I take a gloomy view of things	.54	.38	.44
9	I am often in a bad mood	.57	.40	.49
12	I often find myself worrying about something	.61	.04	.37
13	I am often down in the dumps	.71	.34	.61
Eigenvalue = 4.9				
Social inhibition				
1	I make contact easily when I meet people	.05	−.70	.50
3	When I meet a lot of people, I get nervous	.36	.54	.42
6	I often feel inhibited in social interactions	.30	.64	.50
8	I find it hard to start a conversation	.15	.75	.58
10	I am a closed kind of person	.17	.65	.45
11	I would rather keep other people at a distance	.08	.59	.36
14	When socializing, I don’t find the right things to talk about	0.08	.74	.55
Eigenvalue = 1.9				

A CFA of the two-factor structure of the DS14-RU indicated a good model fit. For the 2-factor solution we found χ^2^/df = 3.2, CFI = 0.93, TLI = 0.91, IFI = 0.93 and RMSEA = 0.067 (90% CI 0.057–0.076), confirming full conformity of theoretical two-factor model with the obtained experimental data.

The construct validity was confirmed using correlation analysis (Table 4). The NA subscale had a positive correlation with the anxiety and depression scale (HADS) and psychosocial stress scale (RSI), as well as with the anger, anxiety and depression as personality traits (STPI). Similar, but less evident, associations were found with the SI scale. The NA and SI scales correlated negatively with the social support scale (MSPSS) and with the curiosity personal trait (STPI).

**Table 4:** Association of negative affectivity and social inhibition with social support, anxiety, depression, stress and anger in Russian respondents

	1	2	3	4	5	6	7	8	9
DS14-RU: Negative affectivity	–								
DS14-RU: Social inhibition	.50**	–							
MSPSS: Social support	−.16**	−.24**	–						
HADS: Anxiety	.47**	.28**	−.19**	–					
HADS: Depression	.32**	.25**	−.30**	.44**	–				
RSI: Stress	.43**	.31**	−.08	.35**	.23**	–			
STPI: Curiosity	−.23**	−.11*	.14**	−.01	−.23**	.05	–		
STPI: Anger	.26**	.15**	−.08	.26**	.20**	.40**	.09	–	
STPI: Trait anxiety	.49**	.33**	−.10*	.53**	.33**	.54**	.01	.45**	–
STPI: Depression	.61**	.36**	−.19**	.44**	.36**	.39**	−.35**	.33**	.62**

*DS14-RU* new Russian version of 14-item Type D Scale, *MSPSS* Multidimensional Scale of Perceived Social Support, *HADS* Hospital Anxiety and Depression Scale, *RSI* Reeder Stress Inventory, *STPI* State-Trait Personality Inventory

*p < 0.05; **p < 0.01

The mean scores for anxiety, depression, psychosocial stress, and anger were significantly higher in the group of patients with Type D personality (Table 5). In addition, patients with Type D personality had significantly lower levels of social support and curiosity.

**Table 5:** Mean levels of social support, anxiety, depression and stress in Type D individuals as compared to non-Type D individuals

	Type D		Non-Type D		P
	M	SD	M	SD	
MSPSS: Social support	65.9	14.4	72.2	12.9	< 0.001
HADS: Anxiety	8.4	3.2	5.5	3.4	< 0.001
HADS: Depression	6.4	3.3	4.5	3.1	< 0.001
RSI: Stress	1.09	0.63	0.69	0.52	< 0.001
STPI: Curiosity	28.2	5.6	30.7	6.5	0.001
STPI: Anger	15.6	3.7	14.4	3.1	0.001
STPI: Trait anxiety	18.6	3.5	15.8	3.3	< 0.001
STPI: Depression	19.4	3.9	16.0	3.7	< 0.001

*М* mean, *SD* standard deviation, *MSPSS* Multidimensional Scale of Perceived Social Support, *HADS* Hospital Anxiety and Depression Scale, *RSI* Reeder Stress Inventory, *STPI* State-Trait Personality Inventory

## Discussion

According to our study, the prevalence of Type D personality, identified with the DS14-RU, was 21.4% among the patients with CVD, and 20.0% among relatively healthy participants, which is somewhat different from the literature regarding patients with CVD. In some studies, the prevalence of Type D personality among the patients with CVD ranged from 23 to 53%, and among healthy individuals in different populations from 13 to 38% [2]. In another Russian study Type D personality was found in 19.3% of patients with atherosclerotic lesions of different localization, which is comparable with our data [33]. Some studies have demonstrated more frequent identification of the Type D personality in patients with CVD than in healthy participants [34]. However, in a Chinese study the prevalence of Type D personality was similar both among patients with CAD (31.4%) and among healthy subjects (31.9%) [35]. In a German study, on the contrary, the prevalence of Type D personality was lower among cardiac patients (25%) than among healthy workers (32.5%) [36]. These conflicting results could be explained by differences in sex and age composition of the different study populations, as well as unaccounted influence of related CVD risk factors such as alcohol consumption, diabetes mellitus, obesity and the impact of other psychosocial factors.

In an Ukrainian study, the CITC value for the original item #3 was .20, and this item had a relatively low factor loadings (.46) [37]. Asian authors also noted that item #3 had a low CITC value (<.40) and, therefore, removal of this item from the questionnaire increased Cronbach’s α of the SI subscale [13]. Bai et al. also reported a low factor loading of item #3 (.34) for their Chinese version of the SI scale [15]. These problematic psychometric characteristics of item #3 in some countries may be due to translation complexity, as well as cultural differences in “*talking to strangers*” as compared to some Western countries [13, 15]. In our study, we faced the same problem and this apparently means that most Russian individuals are not used to talk with strangers. The replacement of the third question «I often talk to strangers» by the alternative item “*When I meet a lot of people, I get nervous*” led to an increase of the internal consistency of the SI subscale of the DS14-RU. This new item loaded .51 on the SI factor of the DS14-RU, but also had a cross-factor loading of .38 on the NA factor, which indicates that this item also partly reflects some elements of NA. However, some other studies also included the item “*When I meet a lot of people, I get nervous*” in their cross-cultural validation of the DS14, despite the relatively strong cross-loading of this item [14].

The results of previous studies have shown a positive correlation of the NA and SI subscales with increased levels of anxiety, depression [9], hostility [13], which corresponds well with the results of our study. An inverse correlation was found between the SI and extraversion [8]. Extraversion is characterized by communicability, the need for social contacts and the need to interact with the outside world. Thus, curiosity and social support to some extent reflect extraversion, and should, in theory, be negatively correlated with the SI, which is confirmed by the results of our study.

Our study demonstrated a positive correlation between NA and stress that is completely consistent with the concept of Type D personality as a distressed personality type that tends to experience a high level of psychoemotional stress [2].

The study of Pedersen et al. demonstrated a negative correlation between Type D personality and the subscales of social support [38]. We found the same correlations in our study. Weng et al. demonstrated positive correlative relationships between the NA, SI and the expression of hostility and total scores on hostility, and these correlations were more pronounced for negative affectivity [13]. We observed the same relationships in our study. All these facts confirm the construct validity of the Russian version of the DS14-RU.

Overall, we found clear evidence for the conceptual two-factor structure, internal consistency and construct validity of the DS14-RU. The results of our study confirmed that Russian cardiac patients with a Type D profile experience increased levels of anxiety, depression, and anger, and lower levels of social support. These findings indicate that the DS14-RU can be used as a brief and valid measure in clinical research and practice. In previous studies of Russian cardiac patients, Type D personality has been associated with decreased physical and mental health-related quality of life after coronary artery bypath grafting [39]. Type D personality was also independently associated with a more than 3-fold increase in risk of adverse cardiovascular events in the year following coronary artery bypath grafting in a study of 683 Russian coronary patients [40]. Moreover, Type D personality was associated with a higher incidence of previous myocardial infarction in Russian patients treated with PCI [41]. These findings support the notion that Type D personality is associated with an increased risk of adverse cardiovascular events in the context of the Russian culture. There are number of potential pathways that may explain this increased risk in Type D patients. Type D personality was associated with an unhealthy carbohydrate metabolism in Russian coronary patients [42], and with a higher prevalence of cardiac risk factors, including hypertension, diabetes, overweight, physical inactivity, and smoking in 1610 Russian cardiovascular patients [43].

## Limitations

There were a few limitations to our study. Our samples consisted of cardiac patients that were admitted to a cardiology inpatient department, and relatively healthy volunteers selected from post-graduate students of Tyumen State Medical University; therefore, these findings may not generalize to the broader population of patients with other diseases or to the Russian-speaking population in general.

## Conclusions

Type D personality was associated with higher levels of anxiety, depression, stress, and aggression, and lower levels of social support and curiosity in Russian patients with CVD. In general, the new Russian DS14-RU is consistent with the original version of the DS14 [6] in terms of reliability, internal scale structure, and construct validity of the questionnaire. Therefore, the DS14-RU is a reliable and brief assessment scale that can be easily used for the identification of individuals with Type D personality traits in Russian-speaking populations.

## Abbreviations

CAD: Coronary artery disease
CFA: Confirmatory factor analysis
CFI: Comparative fit index
CITC: Corrected item-total correlations
CVD: Cardiovascular diseases
EFA: Exploratory factor analysis
HADS: Hospital anxiety and depression scale
IFI: Incremental fit index
MIIC: Mean inter-item correlations
MSPSS: Multidimensional scale of perceived social support
NA: Negative affectivity
PCI: Percutaneous coronary intervention
RMSEA: Root mean-square error of approximation
RSI: Reeder stress inventory
SI: Social inhibition
STPI: State-trait personality inventory
TLI: Tucker-Lewis index
